# Recombinant Collagen Engineered to Bind to Discoidin Domain Receptor Functions as a Receptor Inhibitor*

**DOI:** 10.1074/jbc.M115.674507

**Published:** 2015-12-23

**Authors:** Bo An, Vittorio Abbonante, Huifang Xu, Despoina Gavriilidou, Ayumi Yoshizumi, Dominique Bihan, Richard W. Farndale, David L. Kaplan, Alessandra Balduini, Birgit Leitinger, Barbara Brodsky

**Affiliations:** From the ‡ Department of Biomedical Engineering, Tufts University, Medford, Massachusetts 02155,; the § Department of Molecular Medicine, Istituto di Ricerca e Cura a Carattere Scientifico San Matteo Foundation, University of Pavia, 27100 Pavia, Italy,; the ¶ Molecular Medicine Section, National Heart and Lung Institute, Imperial College London, London SW7 2AZ, United Kingdom,; the ‖ Department of Microbiology and Infectious Diseases, Faculty of Medicine, Toho University School of Medicine, Tokyo 143-8540, Japan, and; the ** Department of Biochemistry, University of Cambridge, Cambridge CB2 1QW, United Kingdom

**Keywords:** collagen, inhibition mechanism, peptides, protein chimera, recombinant protein expression, discoidin domain receptor, binding, triple-helix

## Abstract

A bacterial collagen-like protein Scl2 has been developed as a recombinant collagen model system to host human collagen ligand-binding sequences, with the goal of generating biomaterials with selective collagen bioactivities. Defined binding sites in human collagen for integrins, fibronectin, heparin, and MMP-1 have been introduced into the triple-helical domain of the bacterial collagen and led to the expected biological activities. The modular insertion of activities is extended here to the discoidin domain receptors (DDRs), which are collagen-activated receptor tyrosine kinases. Insertion of the DDR-binding sequence from human collagen III into bacterial collagen led to specific receptor binding. However, even at the highest testable concentrations, the construct was unable to stimulate DDR autophosphorylation. The recombinant collagen expressed in *Escherichia coli* does not contain hydroxyproline (Hyp), and complementary synthetic peptide studies showed that replacement of Hyp by Pro at the critical Gly-Val-Met-Gly-Phe-Hyp position decreased the DDR-binding affinity and consequently required a higher concentration for the induction of receptor activation. The ability of the recombinant bacterial collagen to bind the DDRs without inducing kinase activation suggested it could interfere with the interactions between animal collagen and the DDRs, and such an inhibitory role was confirmed *in vitro* and with a cell migration assay. This study illustrates that recombinant collagen can complement synthetic peptides in investigating structure-activity relationships, and this system has the potential for the introduction or inhibition of specific biological activities.

## Introduction

Genomic studies of prokaryotic organisms identified more than 100 genes that encode proteins with collagen-like (Gly-Xaa-Yaa)*_n_* repeating sequences (1). A number of these bacterial collagen-like proteins have been expressed in recombinant systems, and all formed triple-helical structures with stability close to the *T_m_* = 37 °C found for mammalian collagens (2). These proteins lack the post-translational modification of Pro to 4-hydroxyproline (Hyp),^5^ which is essential for triple helix stabilization in animal collagens, and alternative stabilization strategies are utilized (3, 4). Pure bacterial collagen-like proteins can be produced in high yield in a recombinant *Escherichia coli* system where their sequences can be easily modified, making them an attractive source of recombinant collagenous material for bioengineering and biomedical applications (5–7). One collagen-like protein, Scl2 (*Streptococcus* collagen-like protein 2) from the Gram-positive bacterium *Streptococcus pyogenes*, has been extensively characterized (8, 9). The Scl2 collagen domain appears to be biologically inert, making it an excellent “blank slate” framework for the introduction of specific activities found in animal collagens (5, 10, 11). Human collagens are known to interact with cell surface receptors, extracellular matrix proteins, proteoglycans, glycosaminoglycans, and enzymes, and the specific (Gly-Xaa-Yaa)*_n_* sequences responsible for an increasing number of interactions have been determined through protein and synthetic peptide studies (12–17). Identification of specific ligand binding sequences in human collagen presents an opportunity to insert defined biological activities in a stable triple-helical scaffold provided by recombinant bacterial collagens such as Scl2.

The development of a recombinant bacterial collagen system with inserted human bioactivities depends on a modular model of collagen activity, where a specific (Gly-Xaa-Yaa)*_n_*-binding motif is responsible for a given bioactivity. Typically, short sequences of tripeptides (Gly-Xaa-Yaa)*_n_*, where *n* = 2 to 6, define a ligand-binding site, and experiments introducing the sequences for recognizing integrins, fibronectin, and heparin into the Scl2 triple-helical domain have demonstrated the expected biological activity, both in solid state binding assays and cell culture studies (11, 18–20). In addition, insertion of the sequence for the unique human matrix metalloproteinase cleavage site of type III collagen led to specific digestion of the recombinant collagen protein at this site (21). Here, we extend the concept of designing modular recombinant collagen with separate collagen functionalities by introducing a high affinity binding site for the discoidin domain receptors (DDRs), which are collagen-activated receptor tyrosine kinases (22).

The cell surface DDR receptors are widely expressed in human tissues and play key roles in the communication of cells with the extracellular matrix. The DDRs regulate fundamental cellular functions, including cell adhesion, proliferation, and migration (22, 23). The DDR family consists of two closely related receptors, DDR1 and DDR2, that are both activated by a number of different collagen types, in particular fibrillar collagens (24, 25). Both receptors play important roles in embryo development, and alterations in DDR function have been related to organ fibrosis, osteoarthritis, and tumor progression (22, 26, 27). Collagen binding to their discoidin homology domain induces receptor autophosphorylation with slow kinetics (24, 25, 28). DDR binding to collagen requires its presentation as a native triple-helical structure. Studies using synthetic triple-helical collagen-mimetic peptides demonstrated that the major binding site for DDR1 and DDR2 in the interstitial fibrillar collagen types I–III contains an essential GVMGFO motif (where O = Hyp) (29, 30). A crystal structure of the discoidin domain of human DDR2 bound to a triple-helical peptide revealed the DDR-collagen (GVMGFO) interface at atomic level resolution (31). Peptide studies indicated additional binding sites, with sequences other than GVMGFO, in the fibrillar collagens for DDR2 but not DDR1 (29, 30).

The goal of this work was to exploit modular designs of bacterial collagens to study interactions with the DDRs. A human type III collagen sequence containing the GVMGFO-based DDR-binding site, conserved in collagen II and the α1 chain of collagen I, was inserted between two triple-helical domains of the bacterial Scl2 sequence. Solid-phase binding assays demonstrated that this recombinant collagen protein bound to recombinant DDR ectodomains, as expected. However, the engineered bacterial collagen did not induce DDR activation, as assessed by receptor autophosphorylation, suggesting a more complicated story than simple modular activity. The production of a recombinant collagen, which could bind to but not activate DDR receptors, raised the possible utility of such constructs as DDR inhibitors, and this inhibitory effect was demonstrated in both a competitive binding assay as well as in megakaryocyte (Mk) migration assays.

## Experimental Procedures

Chemicals used in all experiments were purchased from Sigma unless otherwise indicated. Collagens type I and type III preparations, provided by Dr. John Ramshaw, were pepsin-extracted from bovine skin as described (32). Hemate P (Aventis-Behring, Milan, Italy), which contains high purity human von Willebrand factor (VWF) and FVIII, was used as-is in the VWF binding assay.

### 

#### 

##### Production of Recombinant Bacterial Collagen Proteins

The protein sequence for the bacterial collagen constructs was based on the original Scl2.28 sequence from *S. pyogenes* (9). The original protein construct with the N-terminal globular domain (V) was modified to include the following: two tandem repeats of the triple-helical domain (CL); an N-terminal hexahistidine tag for purification; and a protease-susceptible sequence (LVPRGSP) between the V and the first CL domains for V domain removal, as described previously (19, 33). Oligonucleotides encoding the type III collagen DDR-binding sequence were designed and synthesized (Invitrogen; sequences provided upon request). These were inserted as annealed dsDNA between the two CL domains of the bacterial collagen constructs through restriction sites XmaI and ApaI included in the oligonucleotide sequences. The final constructs containing the DDR-binding sites were cloned into the pColdIII vector (Takara Bio Inc.) through NdeI and BamHI restriction sites. All enzymes for cloning were purchased from New England Biolabs. DNA sequencing to confirm fidelity was carried out at the Tufts Core Facility. The DNA constructs and their subsequent proteins were denoted as SCl, for *Streptococcus* collagen-like proteins. The recombinant SCl proteins containing DDR-binding sequences were denoted as SCl-DDR_4_ (inserted sequence, GPRGQPGVMGFP), SCl-DDR_6_ (inserted sequence, GPSGPRGQPGVMGFPGPK), and SCl-DDR_6 F-A_ (inserted sequence, GPSGPRGQPGVMGAPGPK), respectively. The bacterial collagen sequences immediately flanking the inserted human DDR-binding sequences were…GKDGQPGKP (Insertion) GPRGEQGPT….

All SCl constructs in the pColdIII vector were expressed in *E. coli* BL21 strain, grown in 20 ml of LB medium with 100 μg/ml ampicillin overnight at 37 °C. This starting culture was used to inoculate 500 ml of LB/ampicillin media in a shaking flask and grown at 37 °C to an *A*_600 nm_ = 0.8. To induce protein expression, 1 mm isopropyl β-d-thiogalactopyranoside (Thermo Scientific) was added to the culture, and the temperature was lowered to 22 °C. After 16 h of induction, cells were harvested by centrifugation and resuspended in His tag column binding buffer (20 mm sodium phosphate buffer, pH 7.4, 500 mm NaCl, 10 mm imidazole) containing 0.25 mg/ml lysozyme and frozen at −80 °C until purification. Purification of the His-tagged SCl proteins was carried out by affinity chromatography with nickel ion binding and imidazole gradient elution on an ÄKTA pure 25L FPLC (GE Healthcare). Frozen cells were thawed and further lysed by sonication. Cellular debris was removed by centrifugation at 8000 × *g* at 4 °C. The supernatant containing the soluble target protein was injected onto binding buffer equilibrated 5-ml HisTrap HP columns (GE Healthcare). The column was then washed sequentially with 3 column volumes of binding buffer, binding buffer plus 50 mm imidazole, and binding buffer plus 100 mm of imidazole. The His-tagged protein was eluted by elution buffer (binding buffer plus 400 mm imidazole). Protein purity was checked by SDS-PAGE (NuPAGE® BisTris 4–12%, Invitrogen). The protein concentration was determined using an extinction coefficient of ϵ_280_ = 9970 m^−1^ cm^−1^ after dialysis into phosphate-buffered saline (PBS, pH 7.4).

##### Production of Recombinant DDR Ectodomain Proteins

The production and purification of recombinant human DDR proteins were as described previously (30). The Fc-tagged DDR1 (DDR1-Fc, with IgG2 sequence) and DDR2 (DDR2-Fc with IgG2 sequence) were isolated from episomally transfected HEK293-EBNA cells. Proteins were purified by affinity chromatography on HiTrap rProtein A column using an ÄKTA^TM^ purifier (GE Healthcare). For some experiments, human DDR1-Fc (with IgG1 sequence) was purchased from R&D Systems (Minneapolis, MN).

##### Peptide Synthesis

The sequences of the peptides used in this study were as follows: GPC(GPP)_5_-GPRGQOGVMGFO-(GPP)_5_GPC-NH_2_; GPC(GPP)_5_-GPRGQPGVMGFO-(GPP)_5_GPC-NH_2_; GPC(GPP)_5_-GPRGQOGVMGFP-(GPP)_5_GPC-NH_2_; GPC(GPP)_5_-GPSGPRGQOGVMGFO-(GPP)_5_GPC-NH_2_; GPC(GPP)_5_-GPSGARGQOGVMGFO-(GPP)_5_GPC-NH_2_; GPC(GPP)_5_-GPSGPRGQAGVMGFO-(GPP)_5_GPC-NH_2;_ GPC(GPP)_5_-GPSGPRGQOGVMGFA-(GPP)_5_GPC-NH_2_; and GPC(GPP)_10_-GPC-NH_2_. Peptides were synthesized by Fmoc (*N*-(9-fluorenyl)methoxycarbonyl) chemistry as C-terminal amides on TentaGel R RAM resin in either an Applied Biosystems Pioneer or a microwave-assisted CEM Liberty Discover automated synthesizer, and purified as described (34). Peptides were verified by mass spectrometry and shown to adopt triple-helical conformation by polarimetry.

##### Mass Spectrometry

Matrix-assisted laser desorption/ionization time of flight mass spectrometry (MALDI-TOF MS) was performed on a Microflex LT system (Bruker Corp., Billerica, MA) with 50% laser intensity using standard LP (linear positive) 60-kDa mode provided by the software. MALDI matrix was prepared by making a saturated sinapinic acid solution in 50% (v/v) acetonitrile and 0.3% (v/v) trifluoroacetic acid. A 6-μl aliquot of 1 μg/ml sample was mixed with 24 μl of matrix, and 1 μl of this solution was plated onto a 96 spot target plate and allowed to dry before spectra acquisition.

##### Trypsin Digestion

Purified SCl proteins in PBS buffer were incubated with 0.01 mg/ml (430 nm) trypsin at 25 °C for 120 min. The reaction was stopped by addition of phenylmethylsulfonyl fluoride (PMSF) to 1 mm. Cleavage was visualized by SDS-PAGE.

##### Circular Dichroism

CD spectra were obtained on AVIV model 420 CD spectrometer (AVIV Biomedical, Lakewood, NJ) using glass cuvettes with a 1-mm path length. Protein solutions were equilibrated for at least 24 h at 4 °C before measurement. Wavelength scans were collected from 190 to 260 nm in 0.5-nm steps with a 4-s averaging time, 1.0 nm bandwidth, and repeated three times. Temperature scans were monitored from 15 to 70 °C at 225 nm with a 10-s averaging time and a 1.5-nm bandwidth. Samples were equilibrated for 2 min at each temperature, and the temperature was increased at an average rate of 0.1 °C/min.

##### Differential Scanning Calorimetry

Differential scanning calorimetry was performed on a NANO DSC II model 6100 (Calorimetry Sciences Corp, Lindon, UT). Each sample was re-dialyzed against PBS overnight before measurement to collect the dialyzed buffer as reference in the experiment. Sample solutions were loaded at 0 °C into the cell and heated at a rate of 1 °C/min until 100 °C.

##### Dynamic Light Scattering

DLS measurements were performed using a DynaPro Titan instrument (Wyatt Technology Corp., Santa Barbara, CA) equipped with a temperature controller using Eppendorf UVette cuvettes with 10-mm path length. Protein concentrations of the samples (in PBS) were adjusted to 1 mg/ml. Samples were centrifuged at 14,000 × *g* for 10 min and filtered through 0.2-μm Whatman Anotop 10 syringe filters before measurement. Samples were measured at 80% laser intensity. Twenty acquisitions were taken for every sample with each acquisition lasting 60 s. To obtain the hydrodynamic radius (*R_h_*), the intensity autocorrelation functions were analyzed by Dynamic software.

##### Solid-phase Binding Assays

The binding of recombinant DDR-Fc proteins to immobilized collagen proteins was measured using a solid-phase binding assay. 50 μl of 200 μg/ml collagen samples in PBS were coated onto Immulon 2HB 96-well assay plates (ThermoFisher Scientific, Waltham, MA) overnight at 4 °C. Denatured samples were first incubated in a 90 °C water bath for 30 min before coating. Plates were washed and blocked by 1 mg/ml BSA in PBS with 0.05% Tween 20 (PBS-T) for 1 h at room temperature. 50 μl of recombinant DDR1-Fc or DDR2-Fc was subsequently added to each collagen-coated well at a concentration of 20 μg/ml (∼220 nm) in PBS and incubated for 3 h at room temperature. For dose-response assays, serial concentrations of DDR-Fc at 60, 20, 6, 2, 0.6, 0.3, 0.15, 0.06, 0.03, 0.015, and 0 μg/ml were used, which correspond to ∼667, 222, 67, 22, 6.7, 3.3, 1.7, 0.7, 0.3, and 0.17 nm DDR-Fc in molar concentration. For competitive binding assays, the plates were coated with 50 μl of 10 μg/ml bovine collagen type III overnight at 4 °C, washed, and blocked as described above. A final concentration of 3 μg/ml (43 nm) DDR-Fc was mixed and incubated with each collagen sample at a final molar concentration of 0, 0.3, 1.3, 5, and 10 μm for 1 h at room temperature. 50 μl of the collagen/DDR-Fc mixtures was subsequently added onto the type III collagen-coated plates and incubated at room temperature for 3 h. Bound DDR-Fc on the plates was detected by incubating first with primary antibody as follows: mouse anti-human IgG1 Fc mAb (Clone 97924, R&D Systems, Minneapolis, MN) at 1 μg/ml; and then secondary antibody as follows: anti-mouse HRP antibody (Santa Cruz Biotechnology, Dallas, TX) at 1:2,000 dilution, each for 1 h. Finally, 50 μl of 3,3′,5,5′-tetramethylbenzidine solution (Invitrogen) was added for the colorimetric reaction. Plates were washed with 200 μl of PBS-T 6 times between every step. Color was allowed to develop at room temperature for 5 min, and 50 μl of 1 m HCl was added to stop the reaction. *A*_450 nm_ was recorded from the 96-well plate using a Spectra Max M2 plate reader (Molecular Devices, Sunnyvale, CA) for data analysis. Appropriate controls were included using the same setup. The VWF binding assay was carried out using the same mass concentrations as DDR-Fc binding assays. For VWF detection, rabbit anti-VWF N-terminal antibodies (1:1000) and anti-rabbit HRP antibodies (1:5000) (Abcam) were used. Binding of DDR1-Fc and DDR2-Fc to chemically synthesized triple-helical peptides was performed using a similar protocol, as described previously, with detection of bound proteins using anti-human Fc coupled to horseradish peroxidase and *o*-phenylenediamine dihydrochloride as a substrate (29, 30).

##### Collagen-induced DDR Activation Assays

Collagen-induced DDR autophosphorylation in HEK293 cells was performed as described (28–30). Transiently transfected cells were stimulated with collagen I or peptides for the indicated times and temperature, and cell lysates were analyzed by Western blotting with mouse anti-phosphotyrosine monoclonal antibody (clone 4G10 from Upstate Biotechnology), goat anti-DDR2 (AF2538 from R&D Systems), or rabbit anti-DDR1 (SC-532 from Santa Cruz Biotechnology) and appropriate HRP-conjugated secondary antibodies. The DDR activation assay on Mks was performed as follows. Mks were differentiated from cord blood-derived CD34^+^ cells for 13 days in StemSpan medium (Stem Cell, Vancouver, Canada) supplemented with 10 ng/ml thrombopoietin, 10 ng/ml IL-11 (PeproTech, London, UK), 1% l-glutamine, and 1% penicillin/streptomycin as described previously (35). Mks (7 × 10^5^) at day 13 of differentiation were plated on 25 μg/ml type III collagen-coated 6-well plates upon 30 min of incubation with 100 μg/ml (580 nm) of the indicated bacterial collagen. In parallel, unstimulated Mks were plated on uncoated wells as control. After 8 h of incubation at 37 °C in a 5% CO_2_ fully humidified atmosphere, Mks were lysed by scraping the well as described previously (36). Lysates were used for Western blotting analysis of DDR1 activation. Membranes were stained with anti-phospho DDR1 (Tyr-792; Cell Signaling, Danvers, MA) and anti-actin (Sigma, Milan, Italy) to show equal loading.

##### Megakaryocytes Migration Assay

Mk migration and invasion assays were performed as described previously (36). Briefly, 8-μm trans-well migration inserts (Millipore) were coated with 25 μg/ml type III collagen overnight at 4 °C. Mks (25 × 10^3^) were seeded on the upper well in 100 μl of StemSpan and incubated at 37 °C and 5% CO_2_. After 16 h, Mks that had passed through the trans-well to the other side of the filters and in the outer wells, which contained StemSpan medium with 100 ng/ml SDF1-α (PeproTech, London, UK), were recovered and counted under an inverted microscope. Thereafter, the upper side of the filters was carefully washed with cold PBS, and cells remaining on the upper face of the filters were removed with a cotton wool swab. Trans-well filters were fixed in 4% paraformaldehyde for 20 min at room temperature, stained using a monoclonal antibody against CD61 (Santa Cruz Biotechnology) and with Hoechst 33258, cut out with a scalpel, and mounted onto glass slides, putting the lower face on the top. Each experiment was performed in at least triplicates. Data are expressed as numbers of total migrated cells per insert or as percentages of cells related to that of the control. Images were acquired using an Olympus BX51 using ×20/0.5 UPlanF1 objective.

##### Data Analysis

Quantitative analyses were performed in triplicate, with results based on the averages of data points and standard deviations presented as error bars. However, the experiments for the dose-response curves consisted of duplicate or single measurements, to measure all samples on the same 96-well plate. In this case, three independent repeat experiments were performed to confirm the consistency of results. The significance level was determined by *p* value using paired-sample Student's *t* test between the means of two samples. *p* < 0.05 is considered significant. Raw data were processed and plotted in either Origin 6.0 (MicroCal Inc.), Excel 2013 (Microsoft Corp.) or Prism (GraphPad Software, Inc).

## Results

### 

#### 

##### Design and Expression of Recombinant Bacterial Collagen with a DDR-binding Motif

A major DDR-binding site contained in fibrillar collagens I–III is the six amino acid motif GVMGFO, which was identified using triple-helical synthetic collagen-mimetic peptides (29, 30). Although these six amino acids are sufficient for binding recombinant DDR ectodomains, as assessed by solid-phase binding assays, the activation of full-length receptors on the surface of cells requires the presence of additional amino acids N-terminal to the GVMGFO motif; peptides presenting GPRGQOGVMGFO (human type III collagen sequence) induce DDR autophosphorylation with similar kinetics to full-length collagen (29). For this study, we modified streptococcal bacterial collagen Scl2.28, by recombinant DNA technology to insert the DDR-binding sequences from human type III collagen. Sequences from the homotrimeric human α1(III) chain were used because the bacterial system produces homotrimers. The GVMGFO motif is completely conserved among the fibrillar types II and III collagens and the α1 chains of type I, with the surrounding sequences highly conserved (type III sequence, GPRGQOGVMGFO; type II sequence, GARGQOGVMGFO, and sequence of the αI chain of type I collagen GARGQAGVMGFO). Note that formation of Hyp (O) from Pro in the Yaa position of the Gly-Xaa-Yaa triplets is a post-translational modification in animal collagens that does not take place in the bacterial expression system; therefore, GVMGFP rather than GVMGFO is present in the recombinant bacterial protein.

**FIGURE 1.**
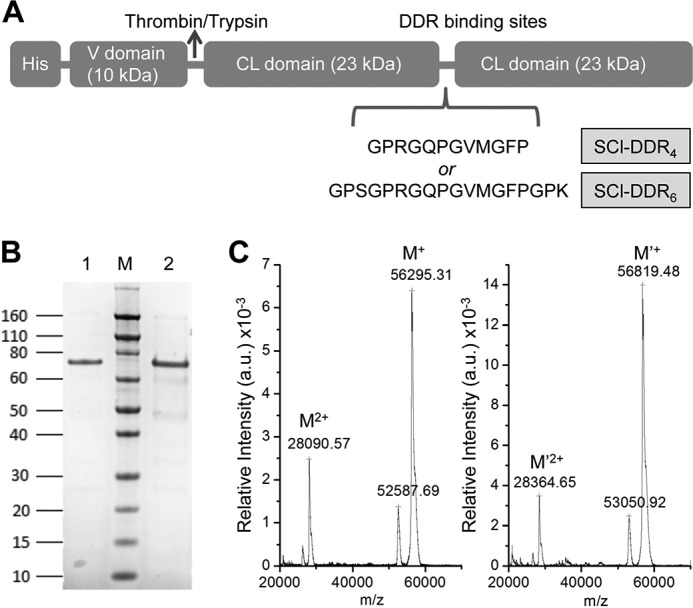
**Production of recombinant collagen-like protein with DDR-binding sequences (SCl-DDR).** *A,* schematic diagram of SCl construct with four or six triplet DDR-binding sequence insertions. *B,* SDS-PAGE showing the purity of the recombinant collagens after His tag column purification. *Lane 1,* SCl-DDR_4_; *lane 2,* SCl-DDR_6_; *lane M,* Novex® Sharp protein standard (Invitrogen); molecular mass is indicated in kDa. Note that collagen proteins always migrate slower in SDS-PAGE than expected on the basis of protein standards. *C,* MALDI-TOF mass spectrometry of SCl-DDR_4_ (*left*) and SCl-DDR_6_ (*right*), showing the molecular mass of the proteins is in good agreement with that expected (Table 1). *a.u.*, arbitrary units.

Two constructs were made containing either four or six triplets of human type III collagen sequence inserted between two tandem CL (Gly-Xaa-Yaa)_79_ domains of bacterial collagen, and these are denoted as SCl-DDR_4_ (GPRGQPGVMGFP) or SCl-DDR_6_ (GPSGPRGQPGVMGFPGPK) (Fig. 1*A*). A specific negative control with a single Phe to Ala replacement was also constructed. Previous peptide studies indicated that replacement of the critical Phe in the GVMGFP motif by Ala eliminated DDR binding and receptor activation (29, 30); therefore, a bacterial protein containing six triplets, including this replacement GPSGPRGQPGVMGAPGPK was also expressed and denoted as SCl-DDR_6 F-A_. All recombinant proteins were expressed in *E. coli* at 22 °C, and the N-terminal His-tagged proteins were purified through a nickel-nitrilotriacetic acid column (IMAC), yielding ∼50 mg/liter of collagen proteins. The purity and identity of the proteins were confirmed by SDS-PAGE and MALDI-TOF (Fig. 1, *B* and *C*; Table 1).

**TABLE 1:** **Characterization of the recombinant bacterial collagen constructs**

	Molecular mass*^a^*		CD		DSC		DLS, *R_h_*	Binding affinity (apparent *K_d_*)*^d^*		
	Calculated	Observed	MRE_200 nm_*^b^*	*T_m_*^CD^	Enthalpy*^c^*	*T_m_*^CAL^		DDR1	DDR2	VWF
	*kDa*	*kDa*		°*C*		°*C*	*nm*	*nm*	*nm*	μ*g/ml*
SCI	55734.39	ND	4677	37.1	4217	38.0	45.1	–	–	–
SCI-DDR_4_	56429.18	56295	3456	36.4	3993	37.0	44.9	53.3 ± 7.1	81.2 ± 17.2	1.2 ± 0.12
SCI-DDR_6_	56952.77	56819	4307	36.6	3818	37.2	42.4	66.0 ± 8.8	69.7 ± 7.4	1.2 ± 0.09
SCI-DDR_6F-A_	56876.67	ND	2913	36.5	ND	ND	ND	–	–	–
Bovine Collage III	ND	ND	ND	ND	ND	ND	ND	15.2 ± 0.9	20.0 ± 1.9	1.0 ± 0.09

*^a^* Calculated mass is based on the amino acid sequence in the open reading frame of each construct. Observed mass is acquired from MALDI-TOF results.

*^b^* Unit for the mean residue ellipticity of the CD is degrees·cm^2^·dmol^−1^.

*^c^* Unit for the calorimetric enthalpy of the DSC is kJ·mol^−1^·K^−1^.

*^d^* The apparent *K_d_* values are calculated based on binding curves obtained from the solid-state binding assays; they are good for internal comparison but do not represent the accurate binding constant between the ligand and receptor. Cells with dash symbols indicate no binding. Cells with ND indicate value not determined for that sample.

##### Structural Characterization of Recombinant Bacterial Collagens

**FIGURE 2.**
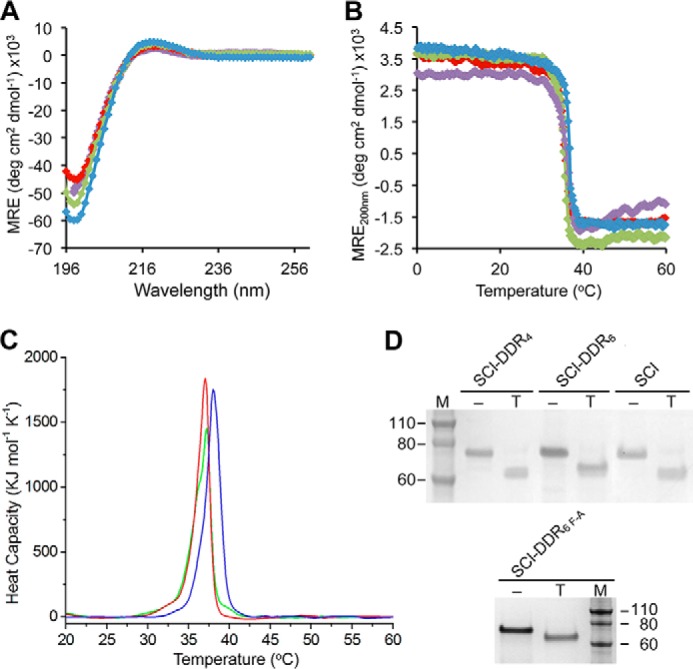
**Structural and thermal analysis of SCl-DDR recombinant collagens.** *A,* circular dichroism wavelength spectra of the recombinant collagens showing triple helix formation. Color code is as follows: SCl (*blue*); SCl-DDR_4_ (*red*); SCl-DDR_6_ (*green*); SCl-DDR_6 F-A_ (*purple*). *B,* circular dichroism temperature scans showing the unfolding profiles of all recombinant collagens. Color code is the same as in *A. C,* differential scanning calorimetry of SCl (*blue*), SCl-DDR_4_ (*red*), and SCl-DDR_6_ (*green*) showing heat capacity as a function of temperature. Areas under the curve indicate the calorimetric enthalpy. *D,* 2-h trypsin digestion (*T*) showing resistance of the triple-helical domain against enzyme digestion. Proteins were analyzed by SDS-PAGE. *Lane M,* Novex® Sharp protein standard (Invitrogen); molecular mass is indicated in kDa. *MRE*, mean residue ellipticity.

Experiments were carried out to confirm that the insertion of the human collagen sequences did not disrupt the triple-helical conformation or stability of the chimeric collagens. The CD spectra of all recombinant proteins showed a characteristic maximum near 220 nm and a minimum near 198 nm, confirming that they adopted a triple-helical conformation (Fig. 2*A*). Monitoring the MRE_220 nm_ *versus* temperature indicated a sharp thermal transition close to 37 °C for all SCl variants (Fig. 2*B* and Table 1), with a very small decrease in *T_m_*^CD^ of ∼0.5 °C for the proteins with insertions *versus* the control. Differential scanning calorimetric data of the samples showed similar pattern of thermal stabilities (Fig. 2*C* and Table 1), with higher *T_m_*^CAL^ observed compared with CD likely due to the 10 times faster heating rate (37). The calorimetry enthalpy of SCl-DDRs also decreased slightly *versus* control but is comparable with other SCl variants with 4–6 triplet human collagen sequence insertions (19).

Trypsin digestion was performed to investigate whether a tight triple helix was maintained at the inserted human sequences. After a 2-h digestion at 25 °C, the globular V domain of SCl was cleaved off, yielding bands with decreased mobility on SDS-PAGE, corresponding to the size of the CL-CL unit (Fig. 2*D*). This indicates that insertions of up to six triplets of DDR-binding site sequences into the CL-CL protein did not alter the triple-helical conformation, as it remained resistant to trypsin digestion. These results confirm that the SCl-DDR_4_, SCl-DDR_6_, and SCl-DDR_6 F-A_ proteins form a typical triple-helical structure.

Dynamic light scattering was used to analyze the particle sizes of the bacterial collagens and to detect potential soluble aggregation of the SCl-DDR solution. The hydrodynamic radii (*R_h_*) of all SCl proteins were ∼44 ± 1.2 nm (Table 1), which is consistent with the previously reported values. These results indicate that proteins with the DDR-binding site insertions did not form aggregates under these conditions.

##### DDR1 and DDR2 Bind to SCl-DDR Proteins

**FIGURE 3.**
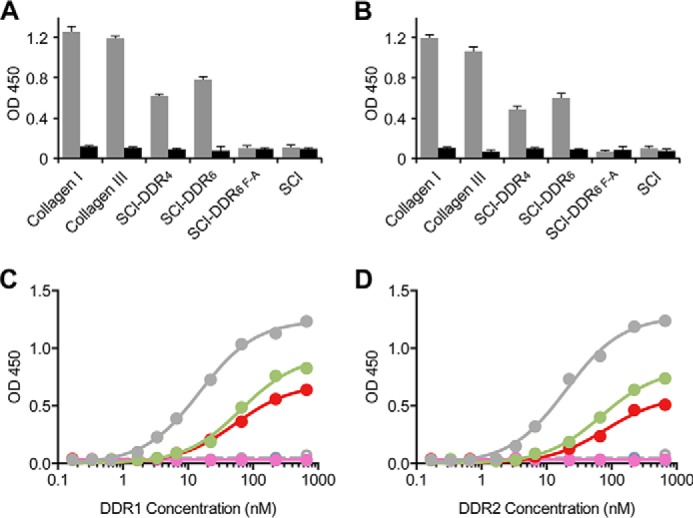
**Solid-phase binding assay of recombinant DDR1-Fc or DDR2-Fc to recombinant SCl-DDR collagens.** *A* and *B,* SCl-DDR constructs in both triple-helical (*gray*) or denatured (*black*) states were immobilized on 96-well plates and incubated with 20 μg/ml (∼220 nm), of DDR1-Fc (*A*) or DDR2-Fc (*B*). Bound proteins were detected with anti-Fc antibodies and measured as absorbance at 450 nm. *C* and *D,* dose response of DDR1-Fc (*C*) and DDR2-Fc (*D*) to collagens adsorbed onto plastic plates. Color code is as follows: collagen type III (*gray*); SCl-DDR_4_ (*red*); SCl-DDR_6_ (*green*); SCl (*blue*); SCl-DDR_6 F-A_ (*purple*); and denatured collagen type III (*open gray symbols*). Data obtained with denatured SCl-DDR_4_ and SCl-DDR_6_ overlap with those of SCl and SCl-DDR_6 F-A_. Data were fitted in GraphPad Prism® using a non-linear fit (one site, specific binding).

Recombinant DDR ectodomain constructs, DDR1-Fc and DDR2-Fc, were used to measure binding to immobilized collagens and SCl-DDR proteins (Fig. 3, *A* and *B*). As expected, the original SCl construct without an insertion did not bind DDR1 or DDR2, whereas the insertion of the four or six tripeptide sequence containing the GVMGFP motif led to DDR binding for both SCl-DDR_4_ and SCl-DDR_6_. The binding signal for these constructs was about 30–50% lower than those seen for the control native type I and type III collagens. DDR binding was seen only when the recombinant proteins were in their native triple-helical state; no binding was seen following denaturation, in agreement with the receptors' binding specificities that are strictly dependent on a native triple-helical conformation (24, 25, 28). The SCl-DDR_6 F-A_ construct showed no binding, consistent with the peptide studies showing Phe is required at the GFP site (29, 30). Initial binding assays were carried out at a DDR concentration of 20 μg/ml (∼220 nm), and this was expanded to obtain dose-response curves using receptor concentrations from 0 to 60 μg/ml (0–667 nm) for DDR1-Fc (Fig. 3*C*) and DDR2-Fc (Fig. 3*D*). SCl-DDR_4_ and SCl-DDR_6_ had similar apparent *K_d_* values for both DDR1-Fc and DDR2-Fc, which were about 3–4 times higher than those for collagen III binding to DDR1-Fc or DDR2-Fc (Table 1), and there was a modest reduction in *B*_max_ (maximal binding) relative to collagen III.

##### SCl-DDR Proteins Are Ligands for von Willebrand Factor

**FIGURE 4.**
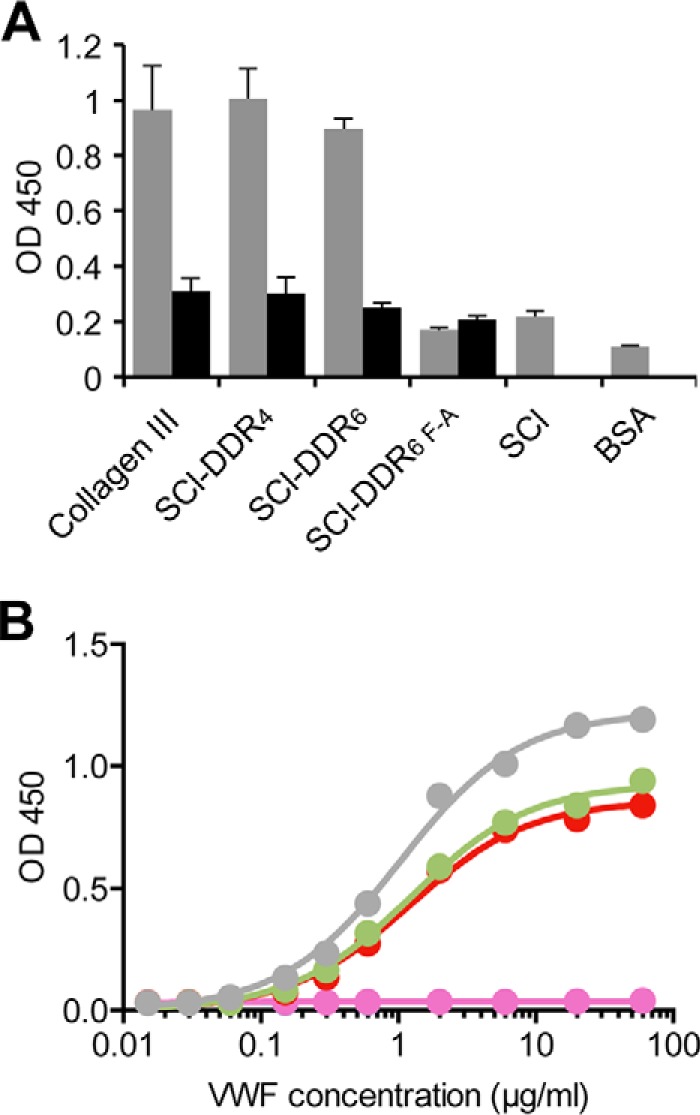
**Solid-phase binding assay of VWF to recombinant SCl-DDR collagens.** *A,* SCl-DDR constructs in both triple-helical (*gray*) or denatured (*black*) states were immobilized on 96-well plates and incubated with 20 μg/ml VWF. *B,* dose response of VWF to collagens adsorbed onto 96-well plates. Bound protein was detected with anti-VWF antibodies and measured as absorbance at 450 nm. Color code is as follows: collagen type III (*gray*); SCl-DDR_4_ (*red*); SCl-DDR_6_ (*green*); SCl (*blue*); SCl-DDR_6 F-A_ (*purple*). Data obtained with denatured collagens are obscured by those obtained with SCl-DDR_6 F-A._ Data were fitted in GraphPad Prism® using a non-linear fit (one site, specific binding).

The DDR-binding motif overlaps with the high affinity-binding site for VWF on collagen type III (RGQOGVMGFO) (38). As this sequence (*i.e.* RGQPGVMGFP, without Hyp) is contained in both SCl-DDR_4_ and SCl-DDR_6_, we tested the proteins for their ability to bind to VWF. Fig. 4 shows dose-dependent binding of VWF to both SCL-DDR_4_ and SCl-DDR_6_. In this case, collagen III, SCl-DDR_4_, and SCl-DDR_6_ did not differ in their affinity to VWF (Table 1). In agreement with the collagen binding specificity of VWF (38), VWF did not bind to denatured collagens or the SCl-DDR_6 F-A_ construct (Fig. 4). This shows that the same sequence GPRGQPGVMGFP in a bacterial collagen context enables binding to VWF as well as to the DDRs, consistent with the peptide findings. These data demonstrate that our engineered SCl proteins have the expected binding characteristics for the inserted binding module.

##### SCl-DDR Proteins Do Not Induce DDR Activation

**FIGURE 5.**
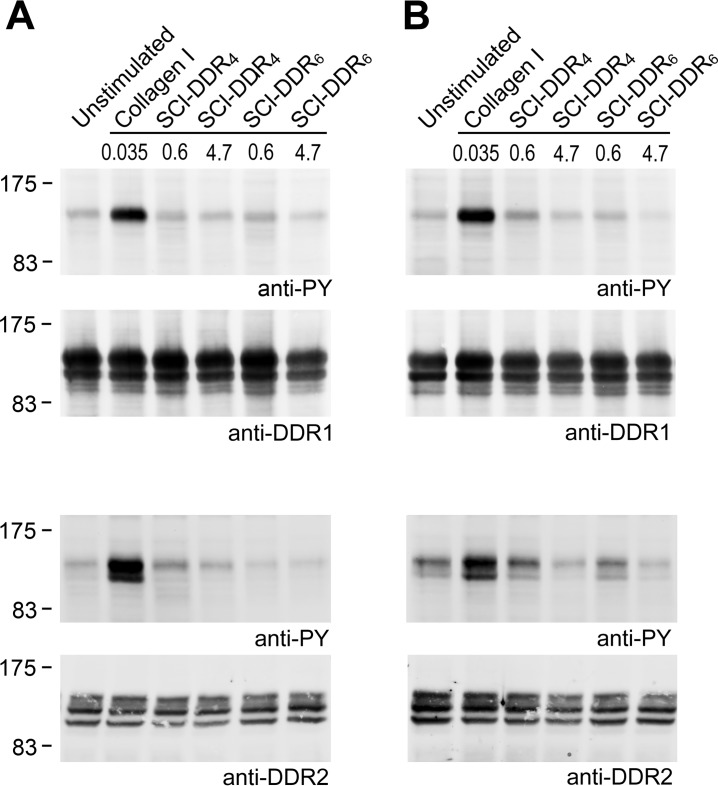
**Recombinant collagens do not induce DDR1 or DDR2 autophosphorylation.** Full-length DDR1 (DDR1a isoform) or DDR2 was transiently expressed in HEK293 cells. After stimulation for 90 min with the indicated collagen samples at 37 °C (*A*) or at 23 °C (*B*), cell lysates were analyzed by SDS-PAGE and Western blotting. Collagen I was used at 10 μg/ml (0.035 μm), which is the concentration previously established to trigger autophosphorylation; SCl-DDRs were assayed at 100 μg/ml (0.6 μm) initially, which is the concentration previously shown to trigger autophosphorylation for collagen peptides, and later at a much higher concentration 800 μg/ml (4.7 μm). For DDR1 samples, the blot was probed with anti-phosphotyrosine mAb 4G10 (*upper panel*), followed by stripping and reprobing with anti-DDR1 (*lower blot*). For DDR2 samples, samples were resolved on two gels. The corresponding blots were probed with anti-phosphotyrosine mAb 4G10 (*upper panel*) or anti-DDR2 (*lower panel*). The positions of molecular mass markers are indicated (in kDa).

Because SCl-DDR_4_ and SCl-DDR_6_ bound to DDR1 and DDR2 in the solid-phase binding assays, experiments were carried out to determine whether these chimeric recombinant constructs could promote receptor activation in HEK293 cells that transiently express full-length DDR1 or DDR2. Collagen binding to the extracellular discoidin homology domain induces activation of the intracellular DDR tyrosine kinase domain, which is manifested by autophosphorylation of cytoplasmic Tyr residues (24, 25). The presence of Tyr phosphorylation was assayed by Western blotting of cell lysates, as described previously (28–30). Collagen type I (at 10 μg/ml or 35 nm)^6^ induced a clear phosphorylation signal for both DDR1 and DDR2, as expected (Fig. 5*A*). In sharp contrast, no DDR activation above background was observed when cells were incubated with either SCl-DDR_4_ or SCl-DDR_6_ using standard incubation conditions at 37 °C. When the initial concentration of 100 μg/ml (0.6 μm) recombinant collagen failed to activate the DDRs, the concentration was increased to the highest testable concentration of 800 μg/ml (∼4.7 μm), but no phosphorylation was observed. Because the triple-helical conformation is required for DDR binding, and activation at 37 °C is close to the melting temperature of the bacterial collagen constructs, it is possible that lack of DDR activation by the bacterial collagen constructs was due to denaturation of the collagens during the 90-min incubation at 37 °C. The activation assays were therefore also carried out at lower temperatures, to avoid possible unfolding of triple helices. Type I collagen induced strong DDR autophosphorylation when cells were incubated at 23 °C, but the bacterial collagen constructs did not induce phosphorylation above background of either DDR1 or DDR2 (Fig. 5*B*). These data suggest that although the SCl-DDR proteins can bind to the DDRs, they do not act as receptor agonists.

##### Effect of Proline Hydroxylation on DDR Binding and Activation

**FIGURE 6.**
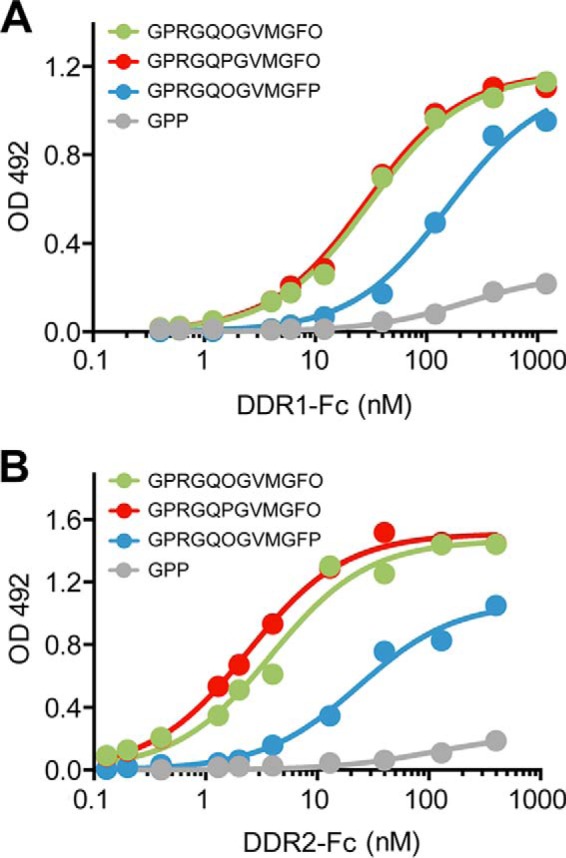
**Solid-phase binding assays of DDR1-Fc or DDR2-Fc to triple-helical collagen-mimetic peptides.** DDR1-Fc (*A*) or DDR2-Fc (*B*) was added for 3 h to 96-well plates coated with peptides at 10 μg/ml. Bound proteins were detected with anti-Fc antibodies and measured as absorbance at 492 nm. Data were fitted in GraphPad Prism® using a non-linear fit (one site, specific binding).

Because the recombinant collagen expressed in *E. coli* is not hydroxylated on proline, it is possible that the absence of Hyp affects DDR binding and receptor activation. Introducing post-translational hydroxylation of Pro in this recombinant bacterial system is complicated, because under aerobic conditions *E. coli* cells do not biosynthesize or transport l-ascorbate into the cytosol (39), which would be required to activate human prolyl-4-hydroxylase introduced through plasmids. Experiments based on a previously published strategy to bypass this requirement (39) have thus far only yielded bacterial collagen with low Hyp levels in our hands. Therefore, experiments to investigate the effect of Hyp on DDR binding and activation were carried out on synthetic triple-helical peptides. Two Hyp are present in the GPRGQOGVMGFO DDR-binding sequence of collagen type III (underlined), and it was previously shown that mutation of the Hyp to Ala in the GQO triplet did not affect the binding affinity to DDR1 or DDR2, although a Hyp to Ala change in the GFO triplet caused a substantial decrease in DDR binding (29, 30). To further investigate whether it is the Pro residue itself or the hydroxylation of the Pro that is critical in the GVMGFO sequence, homologous peptides with GPRGQOGVMGFP and GPRGQOGVMGFO were synthesized and tested. A control peptide with the sequence GPRGQPGVMGFO was also synthesized, which was expected to bind to the DDRs with similar affinity to the native sequence. Solid-phase binding assays showed that the peptide with GPRGQPGVMGFO did indeed bind to DDR1-Fc and DDR2-Fc to similar extents as a peptide with GPRGQOGVMGFO (Fig. 6). In contrast, GPRGQOGVMGFP resulted in weaker binding to DDR1-Fc and DDR2-Fc, compared with the native sequence.

**FIGURE 7.**
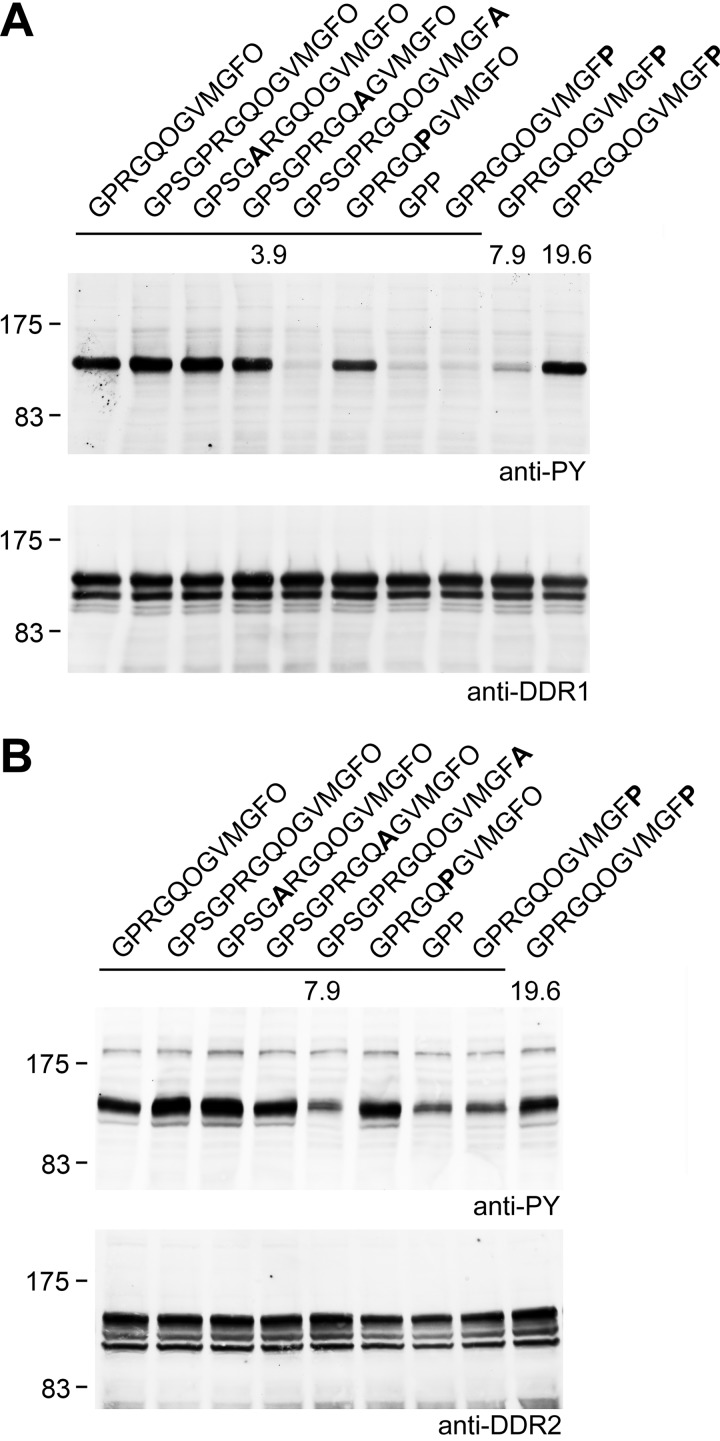
**DDR activation by collagen-mimetic peptides.** Hyp in GVMGFO is important but not essential for DDR1 or DDR2 activation. Full-length DDR1a or DDR2 was transiently expressed in HEK293 cells. After stimulation for 90 min at 37 °C with the indicated peptide samples, cell lysates were analyzed by SDS-PAGE and Western blotting. Peptides were used at 50 μg/ml (3.9 μm) or 100 μg/ml (7.9 μm); the peptide without Hyp was also assayed at 250 μg/ml (19.6 μm). *A,* DDR1 samples, blot was probed with anti-phosphotyrosine mAb 4G10 (*upper panel*), followed by stripping and reprobing with anti-DDR1 (*lower blot*). *B,* DDR2 samples, samples were resolved on two gels. The corresponding blots were probed with anti-phosphotyrosine mAb 4G10 (*upper panel*) or anti-DDR2 (*lower panel*). The positions of molecular mass markers are indicated (in kDa).

The peptides were tested for their ability to induce DDR phosphorylation (Fig. 7). Although the GPRGQPGVMGFO peptide could effectively induce DDR1 or DDR2 phosphorylation when tested at 50 μg/ml (3.9 μm) or 100 μg/ml (7.9 μm), the peptide with GPRGQOGVMGFP required higher concentrations (∼250 μg/ml or 19.6 μm) to stimulate similar levels of DDR1 or DDR2 autophosphorylation. These results are in keeping with the reduced binding affinity of GPRGQOGVMGFP compared with GPRGQOGVMGFO and suggest that the presence of Hyp in GVMGFO, although important for higher affinity DDR binding, is not essential to induce DDR phosphorylation Additional peptides contained Pro to Ala substitutions or Hyp to Ala substitutions in the sequence GPSGPRGQOGVMGFO. Activation of the DDRs by these peptides was as expected; peptides with GPSGARGQOGVMGFO or GPSGPRGQAGVMGFO induced DDR phosphorylation to the same extent as GPSGPRGQOGVMGFO, in line with their ability to bind DDR1 and DDR2 to similar extents as peptides with the native sequence (29, 30). In contrast, a peptide with GPSGPRGQOGVMGFA drastically reduced DDR binding (29, 30) and receptor activation (Fig. 7). Compared with collagen, a much higher molar concentration of these synthetic peptides is required for DDR activation (∼4 μm peptide *versus* 0.03 μm collagen), but the reasons for this are not known (29, 30).

##### Recombinant SCl-DDR as an Inhibitor of DDR Binding and Activation

**FIGURE 8.**
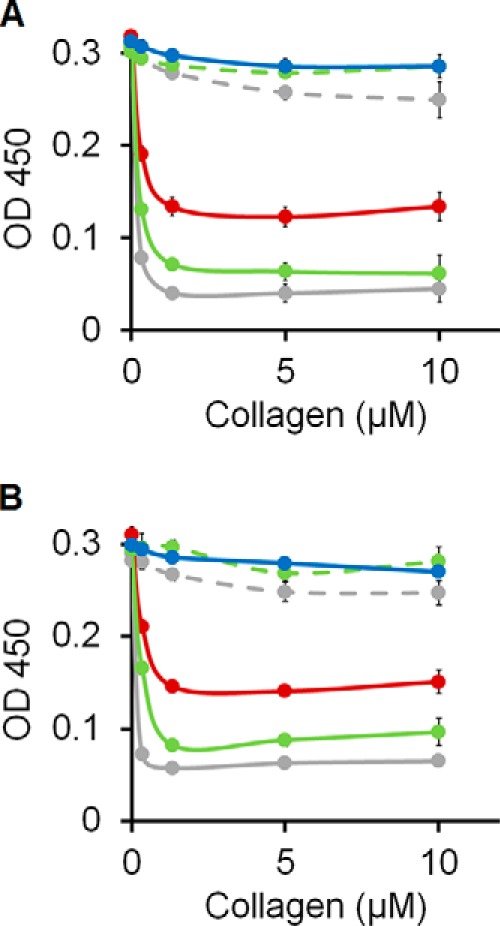
**Competitive inhibition by SCl-DDR of the interactions between collagen type III and recombinant DDR2-Fc.** Collagen type I, SCl-DDR_4_, or SCl-DDR_6_ were incubated with DDR1-Fc (*A*) or DDR2-Fc (*B*) for 1 h at room temperature before addition to plate-immobilized collagen type III. Bound DDR-Fc was detected with anti-Fc antibodies and measured as absorbance at 450 nm. Color code is as follows: collagen type I (*gray*); SCl-DDR_4_ (*red*); SCl-DDR_6_ (*green*); SCl (*blue*). *Dashed lines* show data for the denatured samples.

Given that the recombinant chimeric collagens could bind to DDR1 and DDR2, but not activate them, their role as a potential inhibitor was explored. First, a competitive solid-phase binding assay was performed to test whether the recombinant collagens could inhibit DDR1 or DDR2 binding to immobilized type III collagen (Fig. 8). Denatured collagens as well as the original SCl, with no insertion, showed only negligible inhibition. Efficient inhibition was observed when excessive amounts of native SCl-DDR recombinant collagens were pre-incubated with DDR1-Fc or DDR2-Fc before addition onto type III collagen-coated wells. Maximal inhibition was achieved at ∼1.3 μm (228 μg/ml) of recombinant collagens, giving a molar ratio between dimeric DDR receptors to trimeric recombinant collagen molecules of ∼1:60. Under the same condition, type I collagen acted as a somewhat more potent inhibitor, showing inhibitory effects at lower concentrations. The ability of the recombinant SCl-DDR collagens to inhibit animal collagen binding to DDR suggests potential for inhibition in a biological setting.

**FIGURE 9.**
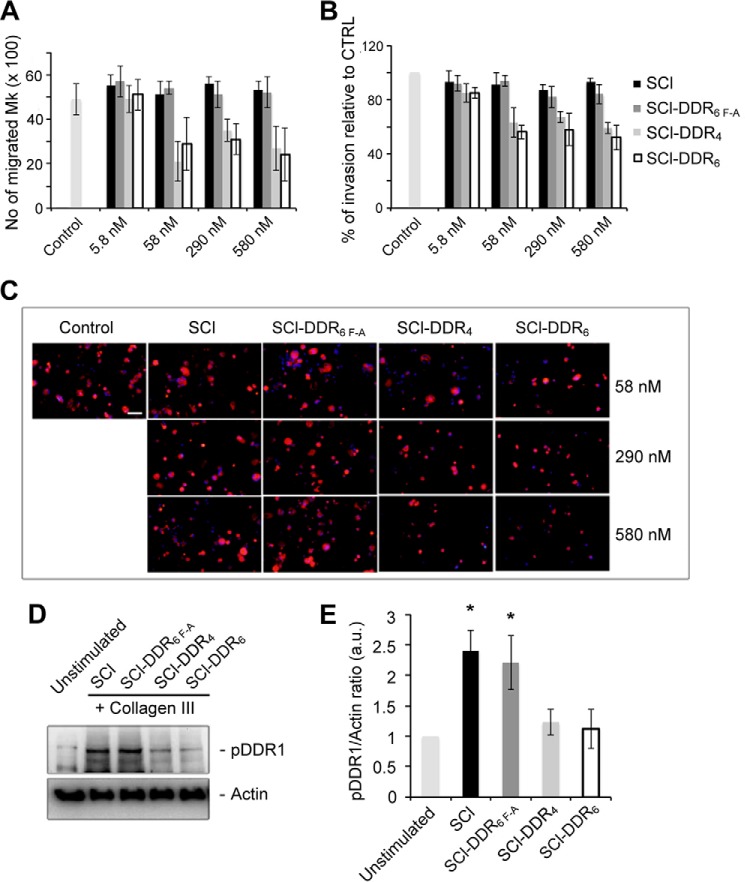
**SCl-DDRs reduce DDR1 activation on type III collagen and result in decreased megakaryocyte migration and invasion.** *A,* Mks were treated with the indicated concentrations of the SCl-DDR recombinant collagens and allowed to migrate in a trans-well migration chamber coated with type III collagen for 16 h. Mks that passed through the filter in the lower chamber were counted. *B,* Mks adhering on the lower side of the Transwell filter were fixed and stained with anti-CD61 antibody and then counted by fluorescence microscopy. *C,* representative images showing Mks that invaded the lower side of the Transwell filter. Cells were stained with anti-CD61 antibody (*red*). Nuclei were counterstained with Hoechst 33288 (*blue*). *Scale bar,* 50 μm. *D,* DDR1 activation assay on Mks stimulated with 100 μg/ml (580 nm) of the indicated SCl-DDR recombinant collagen and plated on type III collagen-coated wells. Unstimulated Mks plated on uncoated wells were used as control. Membranes were probed with the indicated antibodies. *E,* densitometry analysis of pDDR1 Western blots. pDDR1/actin ratio was calculated and shown as relative to unstimulated Mk. Data are presented as means ± S.D. of at least three independent experiments. *Asterisks* indicate *p* < 0.05.

DDR1 is known to support the migration of several types of cells, and the ability of recombinant SCl-DDR_6_ protein to inhibit such migration was investigated using a previously established Mk migration system where DDR1 could be activated by fibrillar collagen, which subsequently promoted cell migration and invasion (36). SCl-DDR was added, at increasing concentrations, into the cell culture media to evaluate whether it could block the binding between Mk surface DDR1 receptors and type III collagen, and thus inhibit cell migration or cell invasion. When SCl-DDR_4_ or SCl-DDR_6_ was added at 10, 50, and 100 μg/ml (equals 58, 290, and 580 nm), fewer cells migrated through the Transwell® membrane, compared with control conditions without adding recombinant collagen (Fig. 9, *A–C*). When the same concentration of SCl without insertion or SCl-DDR_6 F-A_ was added, there was no obvious change in the number of cells migrating through the membrane. Of note, 1 μg/ml (6 nm) of all the bacterial collagens tested did not result in any reduction of Mk migration. The level of phosphorylated DDR1 in the Mks plated on collagen type III was also analyzed by Western blot. In the presence of SCl-DDR_4_ and of SCl-DDR_6_, there was a reduction in the level of phosphorylated DDR1 to a level comparable with unstimulated Mks as shown by the level of phosphorylated DDR1 normalized to actin (Fig. 9, *D* and *E*). SCl or SCl-DDR_6 F-A_ did not affect the level of DDR1 phosphorylation. These results suggest that SCl-DDR_6_ and SCl-DDR_4_ compete with coated type III collagen for binding DDR1 on the surface of Mks, thereby preventing the activation of DDR1 and delaying cell migration in this cell culture system.

## Discussion

We report on a recombinant protein with sequences from the DDR binding region of human collagen type III inserted within a triple-helical bacterial collagen context. Collagen, as the major component of the extracellular matrix, contains more than 50 biologically active sites that interact with other extracellular matrix proteins or cell surface receptors and regulates normal cellular and tissue functions (40). The stringent requirement of forming triple-helical structures complicates the analysis of collagen ligand-binding sites. Early studies used collagen fragments from cyanogen bromide digestion and rotary shadow electron microscopy to identify the rough location of some bioactive sites within collagen. The development of libraries of overlapping triple-helical peptides, the so-called collagen Toolkit peptide libraries, allowed a comprehensive analysis of binding sites within the fibrillar collagens type II and type III (12) and formed the basis for successful definition of the minimal binding sites of an increasing number of key collagen activities, including the DDR-binding sequence used in this study (29, 30).

Although the development of synthetic triple-helical peptides led to major advances in defining collagen binding activities, alternative recombinant strategies are valuable for confirming the peptide results and allowing their application to biomaterials. The use of engineered bacterial collagen-like proteins as a complementary approach allows large scale production of the recombinant protein by standard protein production methods and the creation of bioactive hydrogels (5, 6, 41). To date, several human collagen interaction regions have been inserted within a recombinant bacterial collagen-like protein, including the binding sites for different integrins, the binding site for fibronectin and heparin, and the cleavage site for MMP1 and MMP-13 (11, 18–21). Varying the length of inserted sequences also allowed definition of the minimally required collagen sequence of a given activity in this system. Using the bacterial collagen system raises the possibility of efficient production of “plug and play” recombinant collagen-like proteins containing selected and multiple collagen functions. Here, we extend the concept of designing modular collagen with separate collagen functionalities by creating a group of recombinant collagen-like molecules to target and regulate DDR signaling. Recombinant collagens containing DDR-binding sequences did bind to DDR receptors, as expected, but they differed from mammalian collagens in their weaker binding affinity and their inability to stimulate DDR autophosphorylation.

Non-modified SCl did not bind to the DDRs; binding was dependent on the presence of a DDR-binding sequence and an intact triple helix, as expected from the strict requirement for native triple-helical conformation for the DDR-collagen interaction. Both SCl-DDR_4_ and SCl-DDR_6_ bound DDR1 and DDR2, but there are small differences in the amount of maximal binding and in the level of inhibition. It is not clear whether these differences are significant, but we cannot exclude the possibility that low inhibition of SCl-DDR_4_ points to a secondary binding site in DDR. The dose-response curves of DDR-Fc proteins to SCl constructs showed reduced binding affinity of recombinant SCl-DDR compared with collagens type I and III (Fig. 3). The most obvious difference between animal collagen and recombinant collagen is the presence of hydroxyproline in the former. Our data with synthetic peptides show that Hyp in the six amino acid-binding motif GVMGFO is required for high affinity DDR binding but that substitution of Hyp with Pro in the preceding triplet (GPRGQPGVMGFO context) does not alter binding affinity (Fig. 6). A previous crystal structure of the DDR2 discoidin domain complexed to a collagen-mimetic peptide showed the Hyp in GVMGFO in the leading chain of the triple helix participates in a hydrogen bonding network involving a salt bridge between Arg-105 and Glu-113 of DDR2, whereas the preceding Hyp makes no contact with DDR2 (31). It is therefore not surprising that mutation to GVMGFP reduces DDR binding, although the GPRGQPGVMGFP substitution has no effect on the DDR-SCl interaction. The presence of Hyp in the GVMGFO motif is thus critical for high affinity DDR-collagen interactions.

In contrast, for VWF, it was previously shown that substitution of Hyp with Ala (GVMGFA context) in a synthetic triple-helical peptide did not affect VWF binding (38), indicating that Hyp in the GVMGFO motif is not involved in binding to VWF. Consistent with this notion, our SCl-DDR constructs bound to VWF with similar affinity to that seen for type III collagen (Fig. 4), which likely reflects the lack of involvement of Hyp in VWF binding.

All previous studies found a correlation between the ability of different collagen types to bind the DDRs and their ability to induce receptor activation, as assessed by autophosphorylation of cytoplasmic tyrosine residues (24, 25, 28, 42, 43). This correlation is extended to collagen-mimetic peptides (Fig. 7) (present study and Refs. 29, 30). It was therefore surprising to see no receptor activation by the recombinant SCl-DDR proteins, despite their ability to bind to the DDRs in a specific manner. The inability to induce transmembrane signaling cannot be explained by denaturation of the SCl-DDR triple helix, because no phosphorylation was observed at 23 °C as well as at 37 °C, although control animal collagens led to a strong phosphorylation signal at both temperatures. Although a lack of hydroxylation in GPRGQOGVMGFO reduced binding affinity, peptide studies showed that hydroxylation was not essential for inducing receptor activation. Higher concentrations of GPRGQOGVMGFP peptide were required for DDR activation, compared with the GPRGQOGVMGFO peptide, but the relative peptide concentrations required for activation were in line with their relative affinities.

Because of the reduced binding affinity of the recombinant collagens compared with animal collagens, higher concentrations of SCl-DDR proteins were tested for DDR activation, but no significant autophosphorylation was observed, even at 800 μg/ml (4.7 μm), the highest concentration that was practical in the cell-based assay following the dilution of the protein stock into cell culture medium. Synthetic peptides require much higher concentrations than native collagens to induce DDR phosphorylation (3–4 μm *versus* 0.02–0.03 μm), especially in the absence of Hyp in the GVMGFO essential sequence, but the underlying reasons are not clear (29, 30). We cannot exclude that the failure of our recombinant collagens to induce DDR phosphorylation is due to an inability to test them at high enough concentrations, but the unexpected uncoupling observed between DDR binding and DDR activation may be related to the unusual nature of DDR activation compared with other receptor tyrosine kinases. Typical receptor tyrosine kinases are activated with fast kinetics (seconds to minutes after ligand binding), whereas the DDRs have protracted activation kinetics and require prolonged incubation with collagen for kinase activation (24, 25). Despite a detailed understanding of the DDR1 ectodomain structure and the DDR2-collagen interaction (31, 44), very little is known about the molecular mechanism of how collagen binding to the DDR discoidin domains results in activation of the cytoplasmic kinase domain. In particular, the cellular mechanisms behind the slow kinetics are unclear. DDR1 binding to animal collagen has been shown to cluster the receptor on the cell surface with fast kinetics (45). It is likely that SCl-DDR proteins bind to the DDRs on the cell surface, but perhaps the bacterial collagen cannot cluster the receptors in the required fashion or cannot mediate a “second step” in activation such as the transition from clustered receptors to phosphorylated receptors. Future experiments are required to address whether SCl-DDR proteins lead to DDR1 clustering on the cell surface. It is currently not established whether DDR phosphorylation results from interactions with collagen that has aggregated during the prolonged incubation times necessary to observe receptor phosphorylation. Possible reasons why the SCl-DDR proteins cannot activate DDRs could include the absence of aggregation for the recombinant SCl-DDR protein, which may be required, or the large number of charges in the SCl triple helix, which could disrupt or prevent interactions of the DDRs with molecules on the cell surface, which may be required for a transition to the phosphorylated state.

The observation that SCl-DDR proteins bind to the DDR ectodomain without stimulating receptor transmembrane signaling led to the prediction that they could inhibit collagen-DDR interactions, which was demonstrated *in vitro* and in a cell migration system. The ability of SCl-DDR molecules to interfere with DDR-collagen interactions provides a basis for the generation of recombinant collagen-based biomaterials with DDR inhibitory properties. Recombinant collagen presents an attractive alternative to animal collagens for collagen biomaterial production, allowing standardized, high yield production with easy sequence modification. The recombinant collagen approach has been hindered by the requirement for post-translational modification of Pro to Hyp, an issue that is largely avoided in bacterial collagen SCl which forms a stable triple helix in the absence of Hyp. Biological activities such as integrin, fibronectin, and heparin binding have been conferred on bacterial collagen through the insertion of modular human collagen sequences. This study thus presents the first example where a modular insertion of the defined DDR-binding sequence did not fully capture biological functionality. However, it showed the potential of inserting a human ligand binding module to inhibit a natural biological activity or process. The generation of recombinant collagen-based biomaterials with DDR inhibitory properties could be useful in the study of the pathological roles of DDR signaling in a number of disease models where DDR signaling is believed to contribute to disease progression (22, 26, 27).

## Author Contributions

B. A., B. L., A. B., and B. B. designed the study and wrote the paper. B. A., V. A., D. L. K., and B. L. analyzed and critically evaluated the results. H. X. and D. G. performed experiments with triple-helical peptides and DDR activation assays in HEK293 cells. V. A. performed the experiments with megakaryocytes. D. B. synthesized the collagen-mimetic peptides. A. Y. performed early cloning experiments. R. W. F. oversaw the peptide design and experiments. All authors read and approved the final version of the manuscript.

## Footnotes

6 The concentrations of animal collagens, the SCl-DDR collagen constructs, and the triple-helical peptides are expressed in terms of molarity of trimer (based on single chain molecular mass of animal collagen at ∼95 kDa, SCl-DDR at ∼57 kDa, and peptide molecular masses depending on their sequence).

## Footnotes

5 The abbreviations used are:

Hyp: 4-hydroxyproline
DDR: discoidin domain receptor
ECM: extracellular matrix
VWF: von Willebrand factor
Mk: megakaryocyte
BisTris: 2-[bis(2-hydroxyethyl)amino]-2-(hydroxymethyl)propane-1,3-diol. Molar concentrations of DDR-Fc listed in this work were single chain concentrations, calculated based on single chain molecular mass of DDR-Fc at ∼90 kDa.
